# Deciphering the Puzzle: Literature Insights on *Chlamydia trachomatis*-Mediated Tumorigenesis, Paving the Way for Future Research

**DOI:** 10.3390/microorganisms12061126

**Published:** 2024-05-31

**Authors:** Rafaela Rodrigues, Carlos Sousa, Nuno Vale

**Affiliations:** 1 PerMed Research Group, Center for Health Technology and Services Research (CINTESIS), Rua Doutor Plácido da Costa, 4200-450 Porto, Portugal; rafaela24sofia@hotmail.com (R.R.); carlos.sousa@unilabs.com (C.S.); 2 CINTESIS@RISE, Faculty of Medicine, University of Porto, Alameda Professor Hernâni Monteiro, 4200-319 Porto, Portugal; 3 Molecular Diagnostics Laboratory, Unilabs Portugal, Centro Empresarial Lionesa Porto, Rua Lionesa, 4465-671 Leça do Balio, Portugal; 4 Department of Community Medicine, Health Information and Decision (MEDCIDS), Faculty of Medicine, University of Porto, Rua Doutor Plácido da Costa, 4200-450 Porto, Portugal

**Keywords:** bacteria-induced oncogenesis, *Chlamydia trachomatis* genital infections, biomarkers, immune response, inflammation, personalized medicine, in vitro approaches, cell–cell communication

## Abstract

Some infectious agents have the potential to cause specific modifications in the cellular microenvironment that could be propitious to the carcinogenesis process. Currently, there are specific viruses and bacteria, such as human papillomavirus (HPV) and *Helicobacter pylori*, that are well established as risk factors for neoplasia. *Chlamydia trachomatis* (CT) infections are one of the most common bacterial sexually transmitted infections worldwide, and recent European data confirmed a continuous rise across Europe. The infection is often asymptomatic in both sexes, requiring a screening program for early detection. Notwithstanding, not all countries in Europe have it. *Chlamydia trachomatis* can cause chronic and persistent infections, resulting in inflammation, and there are plausible biological mechanisms that link the genital infection with tumorigenesis. Herein, we aimed to understand the epidemiological and biological plausibility of CT genital infections causing endometrial, ovarian, and cervical tumors. Also, we covered some of the best suitable in vitro techniques that could be used to study this potential association. In addition, we defend the point of view of a personalized medicine strategy to treat those patients through the discovery of some biomarkers that could allow it. This review supports the need for the development of further fundamental studies in this area, in order to investigate and establish the role of chlamydial genital infections in oncogenesis.

## 1. Introduction

According to estimates from the World Health Organization (WHO), cancer ranks among the leading causes of morbidity and mortality globally, accounting for about 20 million new cases and 10 million deaths per year [1]. It is also important to highlight that approximately 20% of all tumors in humans are estimated to result from infections caused by specific bacteria and viruses [2,3,4]. In detail, it is widely recognized that high-risk human papillomavirus (HPV) types, such as HPV-16 and HPV-18, have the potential to cause several types of anogenital and head and neck cancers, especially, the most well-established form, cervical cancer [5]. HPV stands as the most prevalent sexually transmitted infection worldwide, and in Europe, cervical cancer ranks as the ninth most commonly diagnosed cancer among women. However, this malignancy is preventable through effective screening programs and vaccination [6]. Furthermore, *Helicobacter pylori* chronic infection it is a well-established risk factor for gastric cancer [7]. Gastric cancer is classified based on two main anatomical subtypes, non-cardia and cardia [8]. *H. pylori* is considered as a class I carcinogen, with up to 89% of non-cardia gastric cancer being attributed to chronic infection [9]. Importantly, gastric cancer is the fifth most common cancer worldwide, with a high associated mortality rate [8]. These are examples of infection that drives tumorigenesis. The mechanisms underlying this have been studied and it is well-recognized that inflammation caused by these chronic or persistent infections, has a crucial role, leading to oxidative stress, DNA damage, and genomic instability, activating oncogenesis, suppressing tumor suppressor genes, and interfering with cell mechanisms of DNA repair and apoptosis [10,11]. Indeed, inflammation is a recognized hallmark of cancer, contributing to cancer development and progression, and creating a microenvironment convenient for tumor growth and metastasis [12]. Inflammation can be triggered by different circumstance, including infections, and it involves the recruitment of immune cells, secretion of chemokines and cytokines, and activation of signaling pathways that support tumor growth [13].

*Chlamydia trachomatis* (CT) infections are among the most common sexually transmitted infections globally [14]. They can be transmitted through anal, vaginal, and oral sex, as well as through vertical transmission [15]. Recent ECDC data show evidence regarding the increased numbers of infections, and these numbers are probably underestimated due to the fact that they do not account for the positive results in all countries, but only the ones that come from public health entities [16]. Importantly, CT is an obligate intracellular microorganism that primarily infects epithelial cells in mucosal tissues; nonetheless, it can also infect phagocytes present at the site of infection [17,18]. The most preferred anatomical region of infection is the genital tract, however there are also extragenital infections, and systemic disease with widespread symptoms can occur [19]. In detail, this complication is linked to Lymphogranuloma venereum (LGV) strains of *Chlamydia trachomatis*, namely L1, L2, or L3, which primarily infects the lymphatic tissues causing potentially irreversible sequelae if adequate treatment is not provided [20].

Herein we will focus on chlamydial genital infections, which can manifest as chronic or persistent, with an inflammatory response associated, which may lead to pelvic inflammatory disease [21,22]. Moreover, there is epidemiological evidence suggesting a potential correlation between this infection and some genital neoplasia, specifically cervical, ovarian, and endometrial cancers [23,24,25]. Some researchers maintain that there is a biological plausibility for this association [25,26,27]. Notwithstanding, in the literature there is nowell-established link between CT infection and these genital tumors (Table 1), with some conflicting study findings; thus, this epidemiological suggestion could have other reasoning, indicating the presence of potential bias and confounding factors [4]. Herein, we aimed to contribute to the body of evidence clarifying the association between CT infection and carcinogenesis.

## 2. Microbiota’s Importance in Tumor Microenvironment

It is important to note that a vast diversity of microorganisms inhabit the human body, encompassing viruses, bacteria, archaea, fungi, and protists, which exist in various symbiotic associations extending from commensal to pathogenic, and are a community termed as the microbiota, which populates particular niches [35]. A broader term is the microbiome, which not only includes the microorganisms themselves but also their genetic information and the surrounding microenvironment [36]. Accordingly, this resident microbiota plays important functions in human bodies and its deregulation, known as dysbiosis, is implicated in alterations in the immune system, infection, and inflammatory processes, and can also trigger tumorigenesis [37]. 

In fact, the concept of the tumor microenvironment has evolved, moving from a perspective solely focused on the tumor itself, and the components that sustain its development and progression, to a more comprehensive view, also considering the important role of the microbiome in some circumstances [38,39]. Initially, it was thought that the tumor microenvironment, besides the tumor cells, comprised all the immune cells, chemokines, cytokines, and other molecules or cells present in the local environment. Currently, thanks to the advancements in next-generation sequencing and bioinformatic technologies, researchers have found new evidence regarding the microbiome’s role in modulating the tumor phenotype in some cancers, as well as modulating growth, inflammation, the immune system response, genome instability, and therapy resistance [38,40,41]. In 2022, the hallmarks of cancer were updated by Hanahan adding other factors that could influence the tumor, including polymorphic microbiomes [12]. The intestinal tract microbiome has been the focus of research, evidencing its involvement in tumorigenesis susceptibility, notwithstanding, other microbiome niches constituted by a different diversity of microbial species are key factors in health and disease states [37,40,42]. This growing understanding of the importance of tissue-specific microbiomes is crucial to understanding the tumor entities for a better treatment in the future. Indeed, several studies reported that changes in tissue-specific microbiota are related to malignant transformation in these tissues [38,43,44,45,46]. It was demonstrated that some bacteria, such as *Fusobacterium nucleatum*, interfere with some molecular pathways involved in tumorigenesis, triggering the invasiveness potential of the cells, through modifications in cell–cell adhesion driving the epithelial-to-mesenchymal transition (EMT) process, which represents an important step in some physiological processes and in tumorigenesis [41,47]. In detail, a *Fusobacterium nucleatum* adhesin named FadA can bind to E-cadherin on tumor cells of the gut epithelium, modulating E-cadherin/β-catenin signaling, which results in more available molecules of β-catenin, triggering the EMT process leading to the invasiveness potential of the cells and ultimately increasing some oncogenes expression [48,49]. 

Other researchers have identified mechanisms linking bacterial migration with the formation of a premetastatic niche in the liver in colorectal cancer, which is an initial step of metastasis formation [50]. In addition, in recent years, researchers have uncovered microbial presence not only within organs once believed to be sterile, including the bladder, prostate, and uterus, but also within solid tumors, such as colorectal and ovarian tumors [39,41,51,52]. It is clear that microbiome alterations in the gynecological tract, as in the gastrointestinal tract, could lead to molecular signaling modulation driving inflammatory milieu and triggering oncogenesis [53,54,55].

## 3. *Chlamydia trachomatis*’s Role in Genital Neoplasia

**Figure 1:**
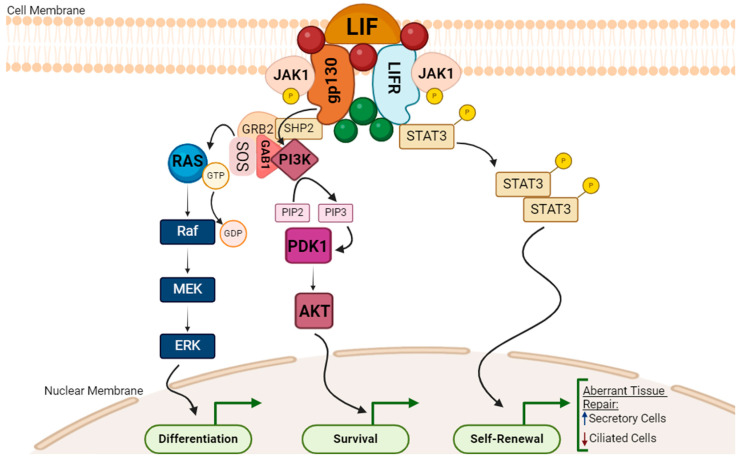
LIF/LIFR signaling pathways potentially involved in reproductive tract microenvironment alterations. The leukemia inhibitor factor (LIF) binds and transactivates the cell–receptor complex of LIFR and gp130 through phosphorylation by JAK1. Then, different signaling pathways could occur, from left to right on the image: The first pathway represented is the MAPK/ERK activation, initiated by a phosphorylation cascade by the gp130/SHP2/GAB1 complex; downstream, RAS-GTP activation culminates in ERK activation, controlling the cellular differentiation process. In parallel, the PI3K/AKT pathway could also be triggered, in which the PI3K mediates the phosphorylation of PIP2 into PIP3, leading to the downstream activation of AKT, involved in cell cycle progression and cell survival. Finally, the last stage is the activation of STAT3 in a cascade of phosphorylation steps, culminating in its translocation to the nucleus and the activation of target genes responsible for self-renewal [61,62]. This altogether, is involved in aberrant tissue repair, resulting in an increase in secretory cells and a decrease in ciliated cells, during CT infection, which contributes to the microenvironment alteration in the female reproductive tract [57]. Figure created using BioRender (accessed on 1 May 2024).

Importantly, it is well-established that chronic infection induced by CT modulates the production of molecules, such as cytokines. In particular, this bacterium could alter the production of the IL-6 molecule family and the leukemia inhibitor factor (LIF), a pleiotropic cytokine, meaning that it couldhave a double function, and pro- and anti-inflammatory roles, depending on the microenvironment context [56,57]. Wang and Wang have studied the molecular signaling pathway involved in chronic CT infection. They found that this molecule is produced by the host to maintain tissue homeostasis and repair tissue; notwithstanding, persistent infection triggers the LIF/LIFR signaling pathways involved in autocrine repair, resulting in a reduction in ciliated cells and an increase in secretory cells (Figure 1) [57,58]. Also, other authors performed in vitro and in vivo studies that corroborate this [59,60]. This milieu triggered by CT is propitious for ectopic pregnancy, infertility and tumorigenesis [57].

In line with this, Siegl and colleagues have investigated the role of an important player in oncogenesis, TP53, which is recognized as the “guardian of the genome”, having tumor suppressor role, since it is responsible for the response to various external factors that could lead to cell stress, such as hypoxia, DNA damage, and many others. In their investigation, they found that CT infection, through the PI3K/AKT molecular signaling pathway, activating HDM2, triggers p53 ubiquitination and subsequent proteasomal degradation, stimulating cell survival [63]. Importantly, these researchers have demonstrated that this p53 inhibition is necessary for CT development because it negatively regulates the pentose phosphate pathway (PPP); therefore, in the absence of the protein, there is no downregulation of glucose-6-P-dehydrogenase (G6PD), allowing DNA damage repair and the production of NADPH, nucleotides, and other optimal metabolites for CT growth by this cellular metabolic pathway. Beyond playing an important role in CT development, it is frequently mutated in cancer; therefore, these alterations in p53 levels, and consequently in its downstream targets, could influence apoptosis, cell senescence, and cell cycle arrest pathways potentially involved in tumorigenesis [64].

**Figure 2:**
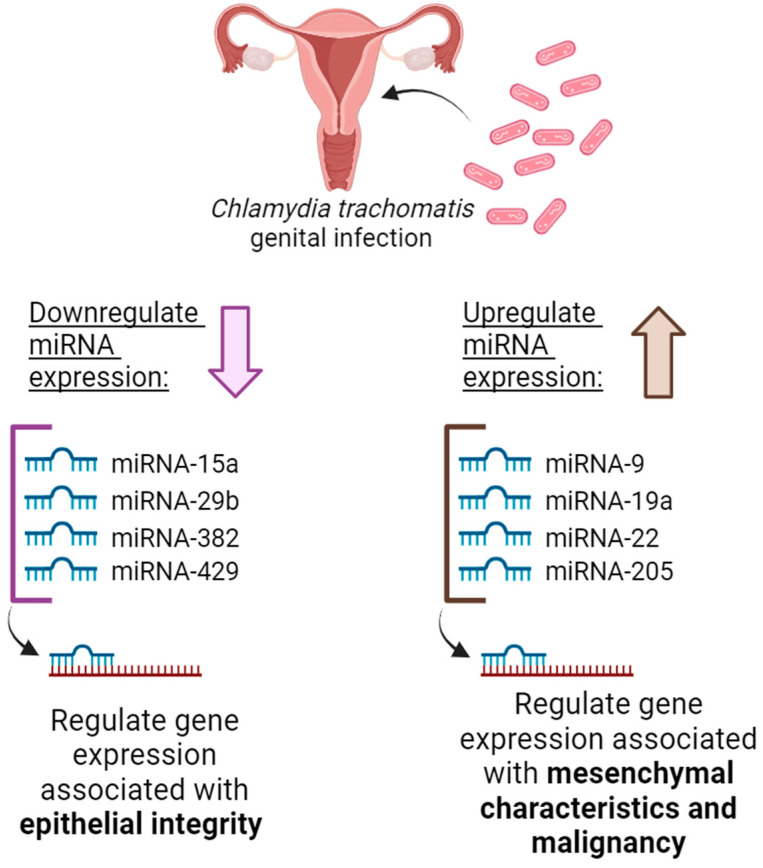
Epigenetic alterations in miRNA expression triggered by CT infection. On the left of the image: CT infection could promote EMT by this epigenetic mechanism in which the bacteria downregulate miRNA expression associated with epithelial integrity, specifically miRNA-15a, miRNA-29b, miRNA-382, and miRNA-429. Additionally, on the right of the image: the bacteria have the potential to upregulate the expression of miRNA-9, miRNA-19a, miRNA-22, and miRNA-205, which are associated with the gene expression regulation of mesenchymal characteristics and malignancy genes [66]. Figure created using BioRender (accessed on 1 May 2024).

Genital tumors have been deeply studied and researchers have found a microbiome signature, namely, *Chlamydia trachomatis* presence in ovarian tumors [42,65]. Igietseme and colleagues have also studied the role of CT genital infections mediating EMT, and they have demonstrated that during infection, these bacteria could dysregulate certain micro-RNAs (mi-RNAs) acting as epigenetic modulators capable of gene expression regulation (Figure 2), specifically downregulating miRNA-15a, miRNA-29b, miRNA-382 and miRNA-429, which are related to =epithelial integrity and contribute to the EMT; in contrast, the bacteria upregulate miRNA-9, miRNA-19a, miRNA-22 and miRNA-205, associated with mesenchymal characteristics and malignant cell transformation [66].

Recently, Horner and colleagues have deeply investigated the CT-induced EMT process, and they maintain that even after the infection resolution, the epigenetic changes triggered by CT may persist in the cells. The authors described the epigenetic modulation triggered by CT, starting from the host cell infection by the infectious elementary body of CT. After the infection, the bacterium starts its replicative cycle of development, releasing bacterial molecules into the cell’s cytoplasm, and modulating DNA transcription. Throughout this process, some cells continue going through the EMT state and others die. In this state, the cells’ profile is a pro-inflammatory and pro-oncogenic one. Concomitantly, CT causes DNA damage with the production of reactive oxygen species (ROS) and the inactivation of DNA repair mechanisms, and the cells could die or preserve this phenotype despite no longer being infected with CT [67]. Additionally, other authors, using ex vivo models, also demonstrate through Phosphoproteome and Transcriptome analysis that CT induces the EMT in host cells [68].

It must be highlighted that as it is well established that CT can cause pelvic inflammatory disease (PID), and PID has been identified as a risk factor for epithelial ovarian cancer, and thus arises the hypothesis thatan association between this bacterium and ovarian tumorigenesis could exist, driven by CT-induced PID; notwithstanding, there is no solid evidence, and further investigation is needed to establish a direct causal relationship [56]. 

Also, it has been proven that *Chlamydia trachomatis* function as a strong cofactor for HPV-related cervical cancer [69,70]. Indeed, CT provides an ideal inflammatory microenvironment for HPV persistence, because this bacterium has the potential to cause cell damage in cervical mucosa and immune escape strategies, contributing to cervical neoplasia development [71,72]. CT has immune evasion mechanisms, in particular, it produces Chlamydial-protease-like activity factor (CPAF), which has an incredible ability to destroy the major histocompatibility complex (MHC), the cellular machinery involved in the adaptative immune response, capable of antigen presentation and subsequent T-cell activation [73]. Besides immunocompromising the host, *Chlamydia trachomatis* could also release exosomes that transport molecules, such as transcription factor 2 and metastasis-associated factor 1, which have the potential to induce angiogenesis, and are also related to ovarian carcinogenesis [74,75]. Additionally, CT is capable of a myriad of other host molecular modulations, such as, histone modifications, DNA repair, ROS formation, driving oncogenic signaling through the Ras-Raf-MEK-ERK pathway, changing the milieu inflammatory profile, tumor suppressor pathways dysregulation and apoptosis inactivation [22,73,76,77,78,79,80]. Altogether, these CT-induced modifications, besides bringing a physiological pro-tumoral milieu, also lead to a favorable environment for HPV and cervical neoplasia development [70,73]. Additionally, following the “state of the art” in CT-induced tumorigenesis mechanisms, the bacterium itself could induce cervical cancer through a pathway involving host cell cycle alterations, specifically modifying centrosome dynamics and cytokinesis, creating a favorable microenvironment for its survival and proliferation [81,82,83]. This has been investigated by Steiert, Faris, and Weber, who stated that intracellular bacteria, such as CT, have the potential to modify cell cycle progression, by altering centrosomes [82]. *Chlamydia trachomatis* has a biphasic developmental cycle constituted by an elementary body (EB) and a reticulate body (RB) [84]. The infectious form, EBs, are incorporated into a membrane-enclosed structure, known as the inclusion, preventing fusion with lysosomes. Rather, it travels along microtubules to the cell’s microtubule-organizing center (MTOC). CT utilizes the cell’s microtubule network to move in the direction of the MTOC. This movement depends on the minus-end-directed motor protein dynein, but remarkably, it does not require the dynactin complex [85]. Importantly, there is evidence demonstrating that bacterial protein synthesis inhibition impairs the bacterium’s capability to reach the MTOC; therefore, C. trachomatis may produce a factor that adjusts dynactin absence. Upon reaching the MTOC, CT inclusion intimately associates with the centrosome, leading to its relocation away from the nucleus, a phenomenon observed throughout the infection cycle [85]. Curiously, along cell division, the chlamydial inclusion is inherited by only one daughter cell alongside the centrosome, while parts of the inclusion membrane stay attached to the centrosome assigned to the uninfected daughter cell [85]. Particularly, Steiert, Faris, and Weber, showed several CT proteins that interact with centriole components and host cell cycle machinery to boost the formation of supernumerary centrosomes and aberrations in cytokinesis: CT850, a protein that associates with centrioles, potentially facilitates the positioning of the CT inclusion at the MTOC. Also, the protein CT223 (IPAM), which interacts with the protein CEP170, is involved in centriole amplification and microtubule disorganization. Additionally, other proteins have been identified: CT847, Dre1 (CT192), CT288, and CPAF, which play specific roles in host cell cycle progression (G1 to S phase), and centriole relocation, blocking multipolar spindle formation. These interactions are crucial for understanding how CT infection increases the risk of cancer, especially cervical cancer, and whether the bacterium acts as a cofactor in HPV-associated tumorigenesis; therefore, future studies are warranted in order to clarify the role of the binding proteins in oncogenesis [82].

## 4. In Vitro Approaches 

As demonstrated throughout this manuscript, *Chlamydia trachomatis* is involved in several potential pathological conditions. Therefore, it is important to perform further studies in this field to better understand microorganisms and host cell interaction. In detail, CT has the potential to invade several cell types, including prostate, testicular Sertoli, cervical, urethral, and conjunctival cells [86,87,88]. In vitro and in vivo studies are the most used strategies that could be used to investigate CT pathogenesis. In vitro studies are easiest to develop because they require fewer ethical considerations, are less expensive, and are faster [89]. To study the cell cycle, 2D-culture of immortalized cells (e.g., HeLa and McCoy cells) is usually used [90,91]. Notwithstanding, this is a simple model of study that does not represent the dynamic and complexity of the in vivo human conditions. The developmental cycle of CT, as an obligate intracellular pathogen, is a complex and dynamic process, taking place within a specific membrane-bound vesicle in the host cell. Within this vesicle, the bacterium varies between two distinct forms, the elementary body (EB) and the reticulate body (RB) [84]. The EB acts as the infectious agent; it is a compacted extracellular form that has nominal metabolic activity. Contrariwise, the RB is metabolically active, and it is responsible for driving intracellular replication. When EBs are internalized, they are transformed into RBs, following rapid binary fission, culminating in replication within 24 h after the infection. Then, the majority of RBs revert to EBs, and finally, mature EBs are released from the host cell, either through cell lysis or extrusion mechanisms, allowing the infection cycle to start in the neighboring cells [19,92,93]. Importantly, during this process, CT has the ability to evade the immune system, culminating in chronic inflammation and leading to cell damage [73]. Therefore, to face this problem, 3D cell culture models, which can better represent a microenvironment, have been implemented, using primary cells [94]. These models encompass scaffold-based 3D cultures, promoting cell-to-extracellular matrix interactions, or non-scaffold 3D cultures, allowing cell-to-cell interactions, as the in vivo conditions [94,95,96]. 

Recently, there have been some developments regarding methods for the co-cultivation of bacteria and spheroids, which is a non-scaffold model, allowing for example the study of the interactions between CT and host cells, from distinct tissues, in a controlled, 3D environment. In line with this, Harimoto and colleagues described a bacterial spheroid co-culture (BSCC) platform, an experimental system designed to investigate the interactions established between bacteria and cancer cells within a 3D culture microenvironment, mimicking the conditions and even the structure of the solid tumors. This protocol is flexible, meaning that it could be applied to multiple bacterial species and cell types, enabling the study of distinct parameters: viability, growth kinetics, and spatial distribution of both bacteria and cancer cells, allowing the understanding of bacteria–tumor interactions [97]. However, as far as we know there are no specific studies regarding the application of this method to CT, which are definitively needed because they could answer many questions about the targeted influence of CT on various cell types and the tumorigenesis process. 

Recently, 3D bioprinting has also been useful in studying CT infection because it is thought to surpass the problem of in vivo models regarding the differences between animals and humans, concomitantly, compared with traditional in vitro approaches, these are better to at mimicking biological conditions [98]. Additionally, the most recent technique is the organ-on-a-chip, which is a complex mechanism, representing the interplay of several organs using biomaterials, allowing us to create a representation of the physiological conditions [99]. This is very interesting because it allows us to represent a tissue response to an infection such as CT infection [94]. Importantly, these methods allow us to gain an insight into the chemical communication mediated by molecules released by the cells, which is very interesting in this topic of research. In line with this, these techniques could allow modifications in cell-surface markers to occur due to the cell–cell communication established, which subsequently could be analyzed in order to characterize the molecular profile of the cells in different cell culture conditions, to understand the cell’s phenotype in different environmental conditions. 

To the best of our knowledge, in the literature, there is a lack of fundamental studies in this field; researchers have not yet studied CT infection using the most developed in vitro strategies. This has particular importance if we aim to understand the hypothetical association between this infection and tumor development or progression, because in line with what was previously stated, to mimic this infection, more developed culture methods must be used in order to achieve conditions closer to the physiological ones, to extrapolate the findings of these studies on preclinical models to the clinical conditions in a more accurate way. The future of CT infection research should encompass the use of more robust in vitro methods to gain a better understanding of CT infection in conditions mimicking the human body.

## 5. *Chlamydia trachomatis* Persistent Infection and Personalized Medicine in Ovarian Cancer

Personalized medicine is a hot topic of current medicine, bringing the opportunity to individualize treatment, choose the best therapeutic approaches based on the characteristics of a particular patient to treat, minimize the unwanted side effects, and enhance treatment effectiveness [100,101]. Currently, we are facing a paradigm shift in patient treatment, moving from a “one size fits all” strategy to personalized treatments. This is particularly evident in oncology and cardiovascular disease therapy, in which the majority of treatments are based on specific biomarkers [102,103]. Also, the presence of certain pathogens could be helpful in guiding personalized cancer treatment, in particular, Holster and colleagues have studied different responses to epithelial ovarian cancer treatment depending on the presence or absence of specific Chlamydia trachomatis infection markers. Interestingly, the authors found that women with epithelial ovarian cancer who had positive serum IgG antibody markers for TroA, HtrA, and MOMP, indicating persistent CT infection, were more likely to respond well to the first-line platinum–taxane treatment compared to those without these antibodies. Moreover, women with positive serum antibody markers for persistent CT infection also had better three-year survival rates compared to those without these markers [56]. Therefore, they concluded that persistent CT infection could lead to chronic inflammation with subsequent damage of the fallopian tubes, which could be related to tumorigenesis of the ovaries, as discussed in the previous section. Finally, they also prove that the immune response of the host may be an important player as a predictor for therapeutic response, as well as better prognosis. Notwithstanding, this study could have potential biases and confounding factors that could influence the conclusions, therefore, it is essential to conduct more fundamental studies in this field to draw further conclusions, gather deep knowledge of the molecular mechanisms underlying this association, and confirm the authors’ hypothesis, in order to translate these findings into concrete benefits for patients.

## 6. Discussion and Conclusions

The number of *Chlamydia trachomatis* infections has been increasing in Europe for years. It is therefore important to understand the pathological mechanisms in order to be able to control and eradicate the infection in the future. Importantly, this infection can be associated with several serious outcomes, including, PID, infertility, and even tumorigenesis. To establish the latter association, epidemiological findings were crucial and paved the way for further research on this topic. In the literature we analyzed several studies with different designs to investigate this hypothetical link. However, we believe that there is a lack of robust studies, with greater reliability and validity, such as prospective studies, which are very difficult to design and conduct in this context, but provided us with the highest level of evidence to establish an association. In addition, studies on endometrial and prostate cancers associated with CT infection are also needed, to clarify the inconsistencies. Baseri and colleagues, and Sfanos and colleagues, argue that CT infection increases the risk of prostate cancer, which is associated with higher PSA levels [28,29]. However, PSA is not a prostate cancer-specific biomarker, which means that higher PSA levels are associated not only with prostate cancer, but also with various other prostate diseases [104]. Gunin et al., have demonstrated that CT infection is related to PID, a condition that increases the risk for endometrial cancer development, and the biological explanation that the authors defend is the fact that pro-inflammatory cytokine production is higher in this context [30]. As we previously stated, CT infections could trigger genital carcinomas and there is a solid suspicion regarding the link of ovarian and cervical cancer with this infection [31,32,34]. Actually, there are conflicting studies in the literature that could have contradictory findings due to some confounding factors or bias involved herein. In detail, differences in sexual behavior can have an influence on both variables, CT infection and tumorigenesis; thus, if the study design does not take this into account, it would introduce bias. Age could be a confounding factor associated with tumorigenesis risk because different age ranges are associated with different risks for CT infection acquisition. Another confounding factor that used to be ignored is smoking, which could also be associated with both variables since it is linked with cervical cancer and with increased risk for CT infection. Bias could also be introduced into studies by socioeconomic status; for example, individuals of lower socioeconomic status do not have access to preventive healthcare, and have increased risk for both conditions. Also, there could be geographical variations in the incidence of these conditions, therefore, studies regarding a certain location are more likely to have conflicting results. Finally, a diagnosis bias could be present due to the differences in the sensitivity and specificity of the diagnostic test used. Therefore, to understand and corroborate this hypothetical association between CT infection and tumorigenesis new techniques must be implemented that allow us to mimic physiological conditions as well as possible, such as non-scaffold 3D cell culture methods. 

In conclusion, the link between *Chlamydia trachomatis* infections and tumorigenesis represents a multifaceted area of research that is not well understood, and needs a more comprehensive study. Our investigation into the tumor microenvironment has denoted the complex interplay between different microbiotas and their influence on tumorigenesis [39,105,106]. In detail, the association between *Chlamydia trachomatis* infections and genital tumors highlights the importance of early detection using screening programs. Understanding the role of CT in this microenvironment opens new avenues for more personalized therapies as demonstrated in ovarian cancer by Holster and colleagues [56]. 

Therefore, we defend that the research on *Chlamydia trachomatis* infections and oncogenesis association not only expands our understanding of tumor microenvironment dynamics and the role of bacterial infection in it, but also paves the way for innovative approaches to prevention, diagnosis, and treatment. 

## Figures and Tables

**Table 1:** Literature evidence linking CTgenital infections with genital tumorigenesis in men and women.

Reference	Field	Study Design	Study Findings
Baseri N. et al., 2021 [28]	Prostate cancer (PCa)	Case–control	Clinical history of CT infection was associated with increased PCa risk.
Sfanos K. et al., 2018 [29]	PCa	Review	CT infection was associated with elevated PSA levels.
Gautam H. et al., 2023 [23]	Endometrial cancer	Case–control	5% of samples tested positive for CT infection in cases; in the control group, no samples were found positive for CT infection.
Gunin A. et al., 2021 [30]	Endometrial cancer	Case–control	Presence of CT in patients with PID have an association with endometrial cancer possibly due to the increased pro-inflammatory cytokine production.
Fortner R. et al., 2019 [31]	Ovarian cancer	Nested case–control	CT seropositivity was associated with higher risk of ovarian cancer.
Idahl A. et al., 2020 [32]	Ovarian cancer	Nested case–control	CT Pgp3 antibodies were associated with mucinous ovarian carcinoma; CT HSP60 antibodies were associated with a higher risk of epithelial ovarian cancer overall and the serous subtype.
Zhu H. et al., 2016 [33]	Cervical cancer	Meta-analysis	CT was significantly linked to increased cervical cancer risk in prospective and retrospective studies.
Li X. et al., 2023 [34]	Cervical cancer	Umbrella Review of Systematic Reviews and meta-analyses	Strong association between CT infection and increased cervical cancer risk.

PCa—prostate cancer; PSA—Prostate-Specific Antigen; CT—*Chlamydia trachomatis*; PID—pelvic inflammatory disease; Pgp3—Plasmid Gene Protein 3; HSP60—Heat Shock Protein 60.

## Data Availability

Not applicable.
